# INCORPORATING SPIRITUALITY INTO COGNITIVE STIMULATION THERAPY GROUPS FOR PERSONS WITH DEMENTIA

**DOI:** 10.1093/geroni/igac059.2804

**Published:** 2022-12-20

**Authors:** Ebow Nketsiah, Max Zubatsky, Marla Berg-Weger

**Affiliations:** Saint Louis University, St. Louis, Missouri, United States; Saint Louis University, St. Louis, Missouri, United States; Saint Louis University - St Louis, MO, Saint Louis, Missouri, United States

## Abstract

With the rise in Dementia-Related Disorders globally, few non-pharmacological approaches have been developed to effectively target cognitive impairment and memory loss. Cognitive Stimulation Therapy (CST) has shown effectiveness in improving cognition and quality of life of older adults with dementia. To date, no studies have incorporated spirituality into CST for improving the cognition and quality of life of older adults with dementia. This study’s aim is to evaluate the effectiveness of CST with a spirituality component compared to the traditional CST group intervention. Our team grouped participants (Nf34) into either spiritual or traditional groups based on their location of residence and level of cognition. Preliminary results showed that spiritual-themed groups improved in mental health status, mobility, and depression. The spiritual groups had a mean difference of 1.85-points improvement on mental status exam as compared to 0.2-point reduction for the traditional groups. In addition, the spiritual groups had a mean difference of 4.80-points improvement in mobility and 0.6-point improvement on depression as compared to a 5.25-points reduction in mobility and a 2.13-points reduction on depression of the traditional groups respectively. Compared to the traditional CST group, there were no significant differences noted regarding cognition and memory recall. Incorporating spirtiuality and other faith-based themes in groups may provide additional benefit for individuals with memory loss. Both healthcare and long-term care facilities may benefit from incorporating group interventions such as CST into their routine care with older adults.

